# Knowledge, Attitude, and Practice of Provincial Dwellers on Prevention and Control of Schistosomiasis: Evidence from a Community-Based Cross-Sectional Study in the Gambia

**DOI:** 10.1155/2020/2653096

**Published:** 2020-06-29

**Authors:** Amadou Barrow, Mansour Badjie, Jainaba Touray, Bakary Kinteh, Musa Nget, Ebrima Touray, Sambou L. S. Kinteh, Saikou Omar Sillah, Lamin Darboe, Yunusa Jallow, Modou Badjan, Modou Gaye, Solomon P. S. Jatta

**Affiliations:** ^1^School of Public Health, Gambia College, Brikama, Gambia; ^2^Department of Public & Environmental Health, School of Medicine & Allied Health Sciences, University of the Gambia, Kanifing, Gambia

## Abstract

**Background:**

Socioeconomically disadvantaged and neglected communities were found to be the most affected groups for schistosomiasis as a result of inadequate safe water and sanitation facilities. In order to inform policies and practices, the present study examined the influence of sociodemographic factors and attitudes on the knowledge and practice in the prevention and control of schistosomiasis in eighteen endemic rural communities in the Gambia.

**Methods:**

In January 2019, a community-based cross-sectional study was conducted in which 383 household heads in rural communities across Kuntaur and Janjanbureh Local Government Areas (LGAs) in Central River Region were recruited. A structured interview questionnaire was developed to elicit information regarding residents' knowledge, attitude, and practice on schistosomiasis prevention and control measures. Percentages, chi-square test, and binary and multiple logistic regression models were used to identify sociodemographic factors associated with the KAP variables. The significance level was set at *p* < 0.05.

**Results:**

Among the 383 participants, only 14.9% had good knowledge, while 54.3% had poor knowledge, 96.9% had positive attitude, and 57.7% had good practice towards prevention and control of schistosomiasis. Older age (≥40 years), compared with residents aged 30–39 years (AOR = 0.331; 95% CI: 0.133, 0.825); ever heard of bilharziasis (AOR = 11.911; 95% CI: 3.452, 41.099); and risks of contact with the polluted river (AOR = 0.101; 95% CI: 0.042, 0.242) were more likely to have good knowledge on schistosomiasis prevention and control in the rural Gambia. Conversely, young people (≤30 years), compared with residents aged ≥40 years (AOR = 2.503; 95% CI = 1.539, 4.071); residents aged 30–39 years (AOR = 2.880; 95% CI = 1.559, 5.320); and male residents (AOR = 2.631; 95% CI = 1.703, 4.067) were more likely to have good practice towards schistosomiasis prevention and control in the rural Gambia.

**Conclusion:**

Despite the low knowledge, rural dwellers' attitudes were found to be positive with slightly good practice towards schistosomiasis prevention and control measures. Thus, while maintaining health system improvement strategies, disease control efforts should focus on these factors as they may influence the knowledge and practices of rural dwellers in a given setting. The findings could prompt appropriate policy responses towards improving the knowledge and practices on schistosomiasis prevention and control in the Gambia.

## 1. Background

Schistosomiasis, widely referred to as bilharzia, continues to be a public health issue in many parts of the world, especially in Africa. It is predicted to cause over 200,000 deaths per annum globally and a minimum of 90% of individuals living in Africa who require treatment [1]. Additionally, estimates show that a minimum of 220.8 million people needed preventive treatment for schistosomiasis in 2017, out of which more than 102.3 million individuals have been said to be treated [2]. Sub-Saharan Africa accounts for over 85% of people living with schistosomiasis in a population that only constitutes 13% of the world's population [3]. Worldwide, there are six species of schistosomes that have an effect on people [4]. In SSA, *S. haematobium* and *S. mansoni* are the most popular species that cause urogenital and intestinal schistosomiasis, respectively [5].

The continuous burden of schistosomiasis in SSA along with its surprising and lifelong consequences may lead to debilitating and permanent clinical complications such as ulceration, blockage of the kidneys, enlargement of the liver and spleen, and barrenness [6]. Children, women, and those who work in contact with natural water bodies tend to be at higher risk across the SSA [7]. Scholars argue that success in schistosomiasis prevention among these groups can lessen the pace of transmission among the population [8]. As a result, their risk of infections was influenced by the poor sanitation status, lack of information, precarious water practices, and negative perspectives about schistosomiasis likewise as low women's educational status [9].

The national prevalence of schistosomiasis (SCH) and soil-transmitted helminthiasis (STH) in the Gambia was 4.3 percent and 2.5 percent, respectively. Schistosomiasis is prevalent in the district of Niani with a prevalence of 22%, while Banjul had the highest prevalence of 55% for soil-transmitted helminthiasis [10]. Schistosoma haematobium is the most dominant parasitic infection in the Gambia. Coendemicity for both types constituted 38% of all districts in the country [10]. Most schistosomiasis educational programs target children who go to school because they are easy to reach, and the degree of such impact varies with the limited continuity of activities over a period of time according to community settings [11].

Population-based health education programs, improved sanitation, and routine mass drug administration remain key-controlled interventions for both SCH and STH [12]. Certain infection control strategies provide access to safe drinking water, proper sanitation, and the promotion of hygiene activities [13]. These interventions are critical in preventing or controlling SCH and STH. Owing to minimal sensitization, the majority of rural dwellers often mixed SCH and STH modes of transmission and prevention. Therefore, it is necessary to combine schistosomiasis and STH prevention measures to ensure that consistent messages about health education are formulated and disseminated. The effectiveness of a community-based control initiative depends on the program's approval by the community [14]. Thus, understanding the knowledge, attitude, and practice (KAP) of rural communities towards SCH and STH is a requirement for quality control program implementation. The functional role of the household head remains very critical at the community level [13,15].

The paper contributes to the knowledge in order to shape policy decisions in the Gambia regarding the involvement of the general population in endemic communities on strategies for prevention and control of schistosomiasis. Therefore, it is important to explore and identify factors influencing communities' knowledge, attitude, and practice on the prevention and control of schistosomiasis as such information is required in the design and implementation of schistosomiasis intervention programs in the Gambia.

## 2. Methods

### 2.1. Study Area

The study was conducted in the Central River Region (CRR) of the Gambia which is divided into two main Local Government Areas (LGAs): Janjanbureh and Kuntaur. Janjanbureh LGA has its administrative headquarters at Janjanbureh, with a population of 126,910. It has a male population of 48.1% (60,001) and a female population of 51.9% (65,909) with a fertility rate of 7.0% [16]. The LGA has five districts, and the major occupations in the region include farming and business. The region has one major health center and four minor health centers. The main referral point for this region is Bansang General Hospital. Kuntaur LGA has its administrative headquarters at Kuntaur, with a population of 99,108. It has a male population of 47.7% (47,233) and a female population of 52.3% (51,875) with a fertility rate of 7.2% [16]. This LGA has five districts, and the major occupations in the region include farming and business. The LGA has one district/major health center and four minor health centers. Bansang General Hospital is the main regional referral point [16].

The Gambia lies within the tropical subhumid ecoclimatic zone, with the rainfall range between 800 and 1200 mm annually [17]. The climate is characterized by two seasons, a wet season (between June and October) and a dry season (November to April), which is six to seven months of no rains [18]. During the dry season, the climate is dominated by dry and dust-laden winds that originate from the Sahara Desert in the northeast [17]. The Central River region has the highest rates of under-five mortality and malnutrition rates and access to improved water sources, and sanitation remains a major challenge [16]. Janjanbureh being an administrative headquarter of CRR that has about 30% of the population is using unimproved sources of water for drinking. The region registered high malnutrition rates among under-five children at 38% as a result of poor access to safe drinking water and sanitation [16]. The literary rate for men and women in CRR is 50.7% and 26.7%, respectively [19]. The net enrolment rate for males was 41.1%, while it was 37.0% for females [20]. The study was conducted in the first half of January 2019.

### 2.2. Study Design, Population, and Participant Selection

The study was a community-based cross-sectional design with a focus on understanding the influence of sociodemographic characteristics and attitudes of rural dwellers' knowledge and practices on the prevention and control of schistosomiasis in CRR. The questionnaire was administered to heads of households of the eighteen identified hotspot communities in the region. A multistage sampling approach was used to select communities in both LGAs within CRR through proportionate sampling; randomization was used to select 18 endemic communities. At the final stage, another proportionate simple random sampling was used to recruit 383 participants from the 18 selected communities. The study involved rural dwellers who were 15 years old and above and willing to participate in the study. However, those who were identified as mentally challenged were exempted from the study.

### 2.3. Sample Size

In estimating the sample size of the study population, the current national prevalence of schistosomiasis of 4.3% was used [10]. The required sample size of 383 household heads was calculated using Dobson's formula as follows. The sample size was calculated using the single proportion formula:

(1) $$\begin{array}{c} n=pq{\left(\frac{z_{\propto /2}}{e}\right)}^{2}, \end{array}$$

where *Z* is the *Z*-value for the 95% confidence interval; that is, alpha = 5% (*z* = 1.96), *p* is the proportion/prevalence of the outcome to be investigated (*p*=0.043), *q* = 1;*p*=0.957, *e* is the precision for the given confidence interval expected expressed as a decimal (*d* = 0.05), and *n* = 64. The computed sample size was adjusted for 10% nonresponses and a design effect of 5.2 which resulted in 383 household heads participating in the survey.

### 2.4. Interview Using the Questionnaire

The interview questionnaire was written in English language and contains both closed and open-ended questions. The draft questionnaire was cast from the English language to core local languages (Mandinka, Fula, and Wolof) and later translated back to the English language in order to ensure consistency and enhance its applicability in various contexts. The tool was pretested for reliability and validity prior to the actual data collection. The validated survey questionnaire sourced information ranging from participants' sociodemographic characteristics, attitudes, practices, and knowledge on the prevention and control of schistosomiasis from all recruited household heads in the study.

### 2.5. Measurement of Outcome Variables

The outcome variables of the study were participants' knowledge and practice towards schistosomiasis. The explanatory variables were selected sociodemographic and attitudinal items. Principal component analysis (PCA) was used to assign the knowledge, practice, and attitude indicator weights. Furthermore, regarding knowledge on schistosomiasis, items used are heard about bilharziasis, source of information, symptoms, causes, and prevention measures. Using PCA, the standardized z-score was used to disentangle the overall assigned scores to poor (<50.0%), fair (50.0%–59.9%), and good (≥60.0%). Regarding practice towards schistosomiasis, items used are urination in water, swimming in the river, boiling of water, the frequent crossing of rivers, open defecation, etc. Using percentage scores for the selected items, the overall assigned scores were classified into good (≥50.0%) and poor practice (<50.0%). Similarly, attitude towards schistosomiasis used the following items: the disease being part of normal growth, toilet utilization, learning the disease at school, antibilharzia deworming utilization, play in the water, etc. Using percentage scores for the selected items, the overall assigned scores were classified into positive (≥50.0%) and negative (<50.0%) attitude.

### 2.6. Ethical Consideration

Prior to the commencement of the study, approval was obtained from the community leaders, Bansang Regional Health Directorate of the Ministry of Health, the Gambia. Ethical clearance for the study was provided by the Gambia College Research Committee. Community visits were made to the various communities in the area, engaging the village heads and village health workers in the communities. The people were sensitized about the nature of the study and study objectives in local languages (Mandinka, Fula, and Wolof). Written informed consent was obtained from each study participant for their enrolment into the study. Participation was entirely voluntary and only those that accepted to participate were recruited for the program.

### 2.7. Data Analysis

The collinearity testing approach adopted the diagnostic analysis to detect interdependence between variables. A cutoff of 0.7 was used to examine the multicollinearity known to cause major concerns . Participants' characteristics were obtained using percentages. The chi-square test was used to examine the association between knowledge, practice, and the explanatory variables. All variables with *p* < 0.20 including significant variables from the bivariate analysis were included in the multivariable logistic regression model to calculate the adjusted odds ratios (AOR) with corresponding 95% CI.

## 3. Results

### 3.1. Characteristics of Residents

A total of 383 residents in the Central River Region were recruited for the study. The sample was proportionately selected across 18 hotspot communities in the region and presented as follows: Kuntaur Fula Kunda (29.2%), Kuntaur (12.3%), Saruja (6.5%), Wassu (13.3%), Bantanto (11.0%), Madina (5.7%), Wellingara (3.7%), Jakaba Manneh Kunda, and Mabally were (2.9%) each; Jahaly Madina (3.4%), Fuladu (2.1%), Ndakaru (1.6%), Boraba (1.8%), Wally Kunda (0.5%), Fula Kunda, and Jahali and Mbari were (0.3%) each.

The mean age of the residents was 33.9 years with a standard deviation of 14.5. Participants below 30 years (47.0%) and 40 years and above (32.9%) accounted for the highest age group in the distribution. There was almost equal representation of gender in the study, while 96.3% were reported to be Muslims. In terms of proximity to the river, 84.9% reported that their homes are close to the river and only 62.9% stated the distance to be within 2 kilometers. Almost half of the residents (49.0%) reported contact with polluted river water with feces/urine as the main risk factor for contracting bilharziasis, followed by 37.1% accounting for those who do not know any of the risk factors for contracting bilharziasis. Slightly more than half of the study participants (57.9%) reported contaminated fecal/urine water/river as a source of bilharziasis. With regard to their opinion on important methods for the prevention of schistosomiasis, 45.3% revealed that they do not know any methods/approaches. Poor appetite (42.1%) and skin infection (42.1%) were reported for the common complications observed as a result of bilharziasis. The use of an herb called “*Sinjango*” and maintaining good hygiene was among the popular approaches for the management of bilharziasis with 83.6% and 81.5%, respectively. At the level of bivariate analysis, residents' characteristics that were found to be significantly associated (*p* < 0.05) with knowledge on schistosomiasis are presented in Table 1.

### 3.2. Multivariable Logistic Regression Model for Predicting Knowledge on Schistosomiasis

The results of predicting residents' knowledge on schistosomiasis prevention and control showed that the likelihood of residents between 30 and 39 years of age decreased significantly by 66.9% to have good knowledge on schistosomiasis prevention and control compared to those of 40 years of age and above who had poor knowledge on schistosomiasis prevention and control (AOR = 0.331; 95% CI: 0.133, 0.825) as shown in Table 2. Residents who had ever heard of bilharziasis were 11.911 times more likely to have good knowledge of schistosomiasis prevention and control compared to those who had never heard of bilharziasis in the category of poor knowledge on schistosomiasis prevention and control (AOR = 11.911; 95% CI: 3.452, 41.099). In addition, residents who opined that contact with the polluted river with feces/urine were less likely to have good knowledge by 89.9% as opposed to those who do not know about the specific risk factors for contracting bilharziasis in the category of poor knowledge on schistosomiasis prevention and control (AOR = 0.101; 95% CI: 0.042, 0.242).

Association of knowledge, attitude, and practice on prevention and control of schistosomiasis in CRR, the Gambia, is shown in Table 3.

As shown in Table 3, the greater proportion of the residents (96.9%) were found to have a positive attitude towards schistosomiasis prevention and control while slightly more than half of the residents (57.7%) adopted good practice towards schistosomiasis prevention and control at the time of the study. There was no statistically significant association between residents' attitude, practice, and knowledge on schistosomiasis prevention and control at (*p*=0.712) and (*p*=270), respectively.

### 3.3. Attitude of Participants towards Schistosomiasis Prevention and Control

Table 4 explores the association between attitude and selected sociodemographic variables, and none were found to be statistically significant.

### 3.4. Participants' Practice towards Schistosomiasis Prevention and Control

In terms of the association between practice towards schistosomiasis with selected sociodemographic variables, age and gender of the residents were found to be statistically significant at (*p*=0.001) and (*p* < 0.001), respectively, as shown in Table 5.

### 3.5. Multivariable Logistic Regression Model for Predicting Practice on Schistosomiasis Prevention and Control

The model (Table 6) predicted that residents below 30 years of age were 2.503 more likely to have good practice towards schistosomiasis prevention and control compared to those of 40 years of age and above (AOR = 2.503; 95% CI = 1.539, 4.071). The likelihood of residents between 30 and 39 years of age increased significantly by the odds of 2.880 to have good practice towards schistosomiasis prevention and control compared to those of 40 years and above (AOR = 2.880; 95% CI = 1.559, 5.320). Male residents were 2.631 times more likely to have good practice towards schistosomiasis prevention and control compared to female residents (AOR = 2.631; 95% CI = 1.703, 4.067).

## 4. Discussion

In the Gambia, this could be the primary study that attempts to demonstrate the impact and nexus of awareness and practices of heads of households with their sociodemographic factors and attitudes regarding the transmission, prevention, and control of schistosomiasis in a scourge rural setting in the Gambia. The findings showed that a larger proportion of household heads/representatives were females, relatively young, and residing within 2 kilometers far away from the river. The results showed more females than males and most people knew something about schistosomiasis with a huge number of them undertaking their water contact activities in unsafe sources of water. While most respondents have heard about schistosomiasis, their awareness of transmission, symptoms, prevention, and control measures remained weak. Rural communities in the Gambia share similar socioeconomic and health profiles; thus, we believe our results are generalizable for the country's entire rural population. More research in other regions should, however, be encouraged.

This study was carried out in endemic areas that are undergoing active surveillance by the Epidemiology and Disease Control Unit of the Ministry of Health, maybe due to the greater proportion of rural dwellers learning about the disease. Furthermore, there were only two MDAs conducted in 2018 using praziquantel and albendazole among children, preschool age, and school-age children from the identified hotspot communities in both Central River Region and Upper River Region of the Gambia. This may explain why very few numbers of participants had good knowledge of the disease. More than half of the participants declared either their experience of the disease through other family members, neighborhood, or personal history of infection, validating the endemicity of these public health problems within these settings. The high burden of the disease among rural dwellers corroborates findings of an earlier study conducted in the Gambia [10]. This calls for more studies among specific vulnerable groups such as women and children from different settings in the Gambia to improve the benefits of targeted schistosomiasis control interventions, particularly education and raising awareness.

Schistosomiasis is progressively widespread in poor rural communities, especially in places where fishing and agricultural activities are predominant. In some countries, the transmission of schistosomiasis may have been hindered through active control programs and/or changing the socioeconomic conditions [21]. Schistosomes are a great public health issue in the Gambia, with an estimated prevalence of 4.3 percent nationally and coendemicity prevalence of 38 percent with soil-transmitted helminthiasis with higher males prevalence than females [10]. For countries like the Gambia and Ethiopia where schistosomiasis is endemic, surveillance is intended towards reducing morbidity and arresting the disease's symptoms. The 2017 Gambian national mapping of schistosomes and STHs assessed variability in water, sanitation, and hygiene (WASH) alongside the parasite infections for school children across the country. The parasitological results showed a prevalence of 4.3% and 2.5% for schistosomiasis and soil-transmitted helminthiasis, respectively [10].

Overall, these findings are in consistent agreement with earlier studies from other schistosomiasis-endemic countries; a high level of schistosomiasis awareness was reported in Yemen [22], Brazil [23], Nigeria [21], and Western Kenya [24]. In comparison, the Malawi [25] and Zimbabwe reported poor awareness of schistosomiasis [26]. A previous study in Senegal reported low awareness of intestinal schistosomiasis among the population after several years of health education at the Ministry of Health interventions using a variety of communication channels like radio, television, and posters [27]. It was proposed that neighbourhoods and schools should be incorporated into health education outreach programs to ensure the fact that the awareness across the community is distributed over a period of time [24].

### 4.1. Study Limitations

Like any other studies, our population of interest were household heads/members either male or female; the implications for study findings generalizability to only household heads, women of childbearing age, or males could be limited. However, the sample size used for the study was computed using the Cochrane formula for descriptive studies. Conversely, the name of a cross-sectional study for causal inferences cannot be made from the results reported. Yet another notable limitation was the lack of adjustment for certain sociodemographic factors.

## 5. Conclusions

The study revealed that information about the cause, transmission, symptoms, prevention, and control of schistosomiasis in rural communities was not sufficient and will be a challenging barrier to the elimination of schistosomiasis from these populations. The reported awareness level about schistosomiasis continues to be very low, and this may mean that a great deal of the government's commitment to schistosomiasis control programmes is extremely necessary. In addition to mass drug administration, school and community health education concerning good personal hygiene and good sanitary practices is crucial among these populations in order to significantly reduce the spread and morbidity of schistosomiasis in rural communities in the Gambia. Continued health education is vital to raising public awareness of schistosomiasis and enables symptomatic individuals to receive treatment with praziquantel. The diagnostic and treatment capacity of government health facilities with regard to schistosomiasis needs to be improved, diagnosis and treatment costs subsidized, alternative water sources provided, and health education programs developed for both community and school settings.

## Figures and Tables

**Table 1:** Participants' knowledge of prevention and control of schistosomiasis in CRR, the Gambia, 2019.

Variable	*n* (%)	Knowledge on schistosomiasis			*p* value
		Good (14.9%)	Fair (30.8%)	Poor (54.3%)	
Age of participants					0.009^*∗*^
Below 30	180 (47.0)	10.0	28.9	61.1	
30–39	77 (20.1)	26.0	32.5	41.6	
40 and above	126 (32.9)	15.1	32.5	52.4	

Gender					0.164
Male	188 (49.1)	16.0	34.6	49.5	
Female	195 (50.9)	13.8	27.2	59.0	

Religion					0.049^*∗*^
Muslim	369 (96.3)	15.4	31.4	53.1	
Christian	14 (3.7)	0.0	14.3	85.7	

River close to your home					0.723
Yes	325 (84.9)	15.4	31.1	53.5	
No	58 (35.1)	12.1	29.3	53.5	

The distance of the river from your home (km) (*n* = 376)			0.007^*∗*^		
2.0 and below	241 (62.9)	13.7	25.7	60.6	
2.1 and above	135 (35.2)	15.6	40.0	44.4	

Ever heard about bilharzia					<0.001^*∗*^
Yes	267 (69.7)	20.2	37.1	42.7	
No	116 (30.3)	2.6	16.4	81.0	

Risk factors for contracting bilharzia					<0.001^*∗*^
Contact with polluted river water with feces/urine	187 (48.8)	23.5	47.1	29.4	
Body contact with an infected person	18 (4.7)	11.1	22.2	66.7	
Walking across water barefooted	15 (3.9)	6.7	40.0	53.3	
Eating unwashed fruits and vegetables	21 (5.5)	9.5	23.8	66.7	
Do not know	142 (37.1)	5.6	10.6	83.8	

Source of bilharzia^*∗∗*^					<0.001^*∗*^
From fecal/urine contaminated water/river	221 (57.9)	20.4	42.5	37.1	
From fecal/urine contaminated soil	64 (16.8)	31.2	32.8	35.9	
From playing in the rainfall	37 (9.7)	21.6	29.7	48.6	
Jumping over a fire	21 (5.5)	33.3	38.1	28.6	
Do not know	121 (31.7)	3.3	5.8	90.9	

Important methods for prevention of bilharzia^*∗∗*^					<0.001^*∗*^
Proper disposal of human waste	75 (20.2)	14.7	33.3	52.0	
Boiling water	80 (21.6)	33.8	41.2	25.0	
Snail eradication	64 (17.3)	32.8	31.2	35.9	
Proper disposal of general waste	42 (11.3)	7.1	45.2	47.6	
Do not know	168 (45.3)	8.3	22.0	69.6	

Complications from bilharzia^*∗∗*^					<0.001^*∗*^
Skin infection	118 (31.1)	22.0	37.3	44.4	
Poor performance at school	99 (26.1)	16.2	39.4	44.4	
Poor appetite	160 (42.1)	19.4	33.8	46.9	
Mood swings	89 (23.4)	20.2	36.0	43.8	
Do not know	72 (18.9)	1.4	12.5	86.1	

How to manage bilharzia^*∗∗*^					0.237
To health-educate people about schistosomiasis	98 (25.6)	13.3	38.8	48.0	
To go to the hospital for treatment	146 (63.7)	19.9	26.7	53.4	
Avoid contact with dirty water	50 (76.8)	10.0	40.0	50.0	
Maintain good personal hygiene	18 (81.5)	16.7	22.2	61.1	
To use a herb called “*Sinjango”*	8 (83.6)	0.0	37.5	62.5	
No response	9 (85.9)	11.1	11.1	77.8	
I do not know or no idea	54 (85.9)	11.1	24.1	64.8	

^*∗*^Statistical significance at *p* < 0.05. ^*∗∗*^Multiple responses.

**Table 2:** Multivariate logistic regression of the factors associated with knowledge on prevention and control of schistosomiasis among study participants at CRR, the Gambia.

Knowledge on schistosomiasis		B coefficient	AOR	95% Cl for OR		*p* value
				LB	UB	
Fair	Intercept	0.689				0.242
	Age of participants					
	Below 30	0.332	1.394	0.632	3.078	0.411
	30–39	−0.569	0.566	0.245	1.306	0.182
	40 and above (ref)	0^b^				
	Ever heard of bilharzia					
	Yes	1.127	3.086	0.859	11.084	0.084
	No (ref)	0^b^				
	Risk factors for contracting bilharzia					
	Contact with polluted river water with feces/urine	0.173	1.188	0.458	3.081	0.722
	Body contact with an infected person	0.033	1.033	0.150	7.098	0.973
	Walking across water barefooted	1.341	3.821	0.377	38.765	0.257
	Eating unwashed fruits and vegetables	0.191	1.211	0.181	8.096	0.843
	Do not know (ref)	0^b^				

Poor	Intercept	2.010				0.000
	Age of participants					
	Below 30	0.370	1.447	0.642	3.261	0.372
	30–39	−1.105	0.331	0.133	0.825	0.018^*∗*^
	40 and above (ref)	0^b^				
	Ever heard of bilharzia					
	Yes	2.477	11.911	3.452	41.099	<0.001^*∗*^
	No (ref)	0^b^				
	Risk factors for contracting bilharzia					
	Contact with polluted river water with feces/urine	−2.295	0.101	0.042	0.242	<0.001^*∗*^
	Body contact with an infected person	−0.771	0.463	0.082	2.596	0.381
	Walking across water barefooted	−0.541	0.582	0.060	5.675	0.641
	Eating unwashed fruits and vegetables	−0.712	0.491	0.085	2.819	0.425
	Do not know (ref)	0^b^				

Model adjusted for participants' sex, the distance of river from home, source of bilharzia, prevention methods of bilharzia, complications, communities, and religion. ^a^The reference category is good knowledge. ^b^This parameter is set to zero because it is redundant. Statistically significant at *p* < 0.05.

**Table 3:** Association between participants' attitude, practice, and knowledge on schistosomiasis prevention and control in CRR, the Gambia, 2019.

Variable	*n* (%)	Knowledge on schistosomiasis			*p* value
		Good (14.9%)	Fair (30.8%)	Poor (54.3%)	
Attitude towards schistosomiasis					0.712
Positive	371 (96.9)	15.1	30.5	54.4	
Negative	12 (3.1)	8.3	41.7	50.0	

Practice towards schistosomiasis					0.270
Good	221 (57.7)	17.2	31.2	51.6	
Poor	162 (42.3)	11.7	30.2	58.0	

**Table 4:** Participants' attitude towards schistosomiasis prevention and control in CRR, the Gambia, 2019.

Variable	*n* (%)	Attitude towards schistosomiasis		*p* value
		Positive (96.9%)	Negative (3.1%)	
Age of participants				0.374
Below 30	180 (47.0)	97.2	2.8	
30–39	77 (20.1)	98.7	1.3	
40 and above	126 (32.9)	95.2	4.8	

Gender				0.515
Male	188 (49.1)	96.3	3.7	
Female	195 (50.9)	97.4	2.6	

Religion				0.493
Muslim	369 (96.3)	96.7	3.3	
Christian	14 (3.7)	100	0	

River close to your home				0.723
Yes	325 (84.9)	96.6	3.4	
No	58 (35.1)	98.3	1.7	

The distance of the river from your home (km) (*n* = 376)				0.504
2.0 and below	241 (62.9)	97.1	2.9	
2.1 and above	135 (35.2)	96.3	3.7	

^*∗*^Statistical significance at *p* < 0.05.

**Table 5:** Participants' practice towards schistosomiasis prevention and control in CRR, the Gambia, 2019.

Variable	*n* (%)	Practice towards schistosomiasis		*p* value
		Good (57.7%)	Poor (42.3%)	
Age of participants				0.001^*∗*^
Below 30	180 (47.0)	63.3	36.7	
30–39	77 (20.1)	66.2	33.8	
40 and above	126 (32.9)	44.4	55.6	

Gender				<0.001^*∗*^
Male	188 (49.1)	68.1	31.9	
Female	195 (50.9)	47.7	52.3	

Religion				0.611
Muslim	369 (96.3)	75.5	42.5	
Christian	14 (3.7)	64.3	35.7	

River close to your home				0.197
Yes	325 (84.9)	59.1	40.9	
No	58 (35.1)	50	50	

The distance of the river from your home (km) (n = 376)				0.813
2.0 and below	241 (62.9)	57.3	42.7	
2.1 and above	135 (35.2)	58.5	41.5	

^*∗*^Statistical significance at *p* < 0.05.

**Table 6:** Binary logistic regression of the factors associated with practice towards schistosomiasis prevention and control among study participants at CRR, the Gambia.

Practice towards schistosomiasis	B coefficient	AOR	95% Cl for OR		*p* value
			LB	UB	
Age of participants					<0.001^*∗*^
Below 30	0.917	2.503	1.539	4.071	<0.001^*∗*^
30–39	1.058	2.880	1.559	5.320	0.001^*∗*^
40 and above (ref)	0				

Gender of the participants					
Male	0.968	2.631	1.703	4.067	<0.001^*∗*^
Female (ref)	0				
Constant	−1.059	0.347			0.002

Model adjusted for the proximity of the river from home. ref = reference category. Statistically significant at *p* < 0.05.

## Data Availability

The data used to support the findings of this study are included in the article. The datasets analyzed during the study are available upon reasonable request from the school administration.

## Abbreviations

CRR: Central River Region
MDA: Mass drug administration
WHO: World Health Organization.
